# Biocontrol Ability and Production of Volatile Organic Compounds as a Potential Mechanism of Action of Olive Endophytes against *Colletotrichum acutatum*

**DOI:** 10.3390/microorganisms10030571

**Published:** 2022-03-06

**Authors:** Yosra Sdiri, Teresa Lopes, Nuno Rodrigues, Kevin Silva, Isabel Rodrigues, José Alberto Pereira, Paula Baptista

**Affiliations:** Centro de Investigação de Montanha (CIMO), Instituto Politécnico de Bragança, Campus de Santa Apolónia, 5300-253 Bragança, Portugal; sdiriyosra4@gmail.com (Y.S.); teresalopes@ipb.pt (T.L.); nunorodrigues@ipb.pt (N.R.); kevin.silva@ipb.pt (K.S.); irodrigues@ipb.pt (I.R.); jpereira@ipb.pt (J.A.P.)

**Keywords:** biocontrol, *Aureobasidium pullulans*, *Sarocladium summerbellii*, anthracnose, volatile compounds

## Abstract

Olive anthracnose, mainly caused by *Colletotrichum acutatum*, is considered a key biotic constraint of the olive crop worldwide. This work aimed to evaluate the ability of the endophytes *Aureobasidium pullulans* and *Sarocladium summerbellii* isolated from olive trees to reduce *C. acutatum* growth and anthracnose symptoms, and to assess *A. pullulans*-mediated changes in olive fruit volatile organic compounds (VOCs) and their consequences on anthracnose development. Among the endophytes tested, only *A. pullulans* significantly reduced the incidence (up to 10-fold) and severity (up to 35-fold) of anthracnose in detached fruits, as well as the growth (up to 1.3-fold), sporulation (up to 5.9-fold) and germination (up to 3.5-fold) of *C. acutatum* in dual culture assays. Gas chromatography–mass spectrometry analysis of olives inoculated with *A. pullulans* + *C. acutatum* and controls (olives inoculated with *C. acutatum*, *A. pullulans* or Tween) led to the identification of 37 VOCs, with alcohols being the most diversified and abundant class. The volatile profile of *A. pullulans* + *C. acutatum* revealed qualitative and quantitative differences from the controls and varied over the time course of microbial interactions. The most significant differences among treatments were observed at a maximal reduction in anthracnose development. At this stage, a set of VOCs, particularly Z-3-hexen-1-ol, benzyl alcohol and nonanal, were highly positively correlated with the *A. pullulans* + *C. acutatum* treatment, suggesting they play a critical role in anthracnose reduction. 6-Methyl-5-hepten-2-one and 2-nonanone were positively associated with the *C. acutatum* treatment and thus likely have a role in pathogen infection.

## 1. Introduction

Olive is an extremely important crop for Mediterranean Basin countries, including Portugal [1]. Olive anthracnose, mainly caused by diverse fungi clustering in the *Colletotrichum acutatum* species complex, is a key constraint to olive production through its effects on olives. The yield loss caused by anthracnose can reach 80–100% in some olive-growing regions of the world [2]. The control of this disease is challenging, being mostly based on the use of copper-based products [2]. Besides its limited efficacy, the use of these products has also led to several environmental problems [3]. Therefore, there is an urgent need to develop more effective and environmentally friendly tools to control anthracnose. This strategy has long been promoted by the EU (Directive 2009/128/EC), and more recently, it has formed part of the European Green Deal, particularly the Farm to Fork Strategy, which aims to reduce pesticide use in the EU by 50% by 2030. One possible approach for meeting this demand is the development of biological control products based on the use of endophytes. There is increasing evidence that this microbial community, living in internal plant tissues, can help host plants deal with pests and diseases using various mechanisms [4]. This beneficial effect is mainly ascribed to the production of secondary metabolites by the endophyte, including volatile organic compounds (VOCs) [4]. Endophyte-derived VOCs are active in disease control as direct antimicrobial agents and as resistance inducers, preventing plant colonization by pathogens [5]. They can also protect plants from pests and diseases by mediating intra- and interspecific interactions in a broad array of organisms [6]. For instance, endophytes can alter the composition of plant volatile organic compounds, making it more attractive to natural enemies of insect pests, and thus minimizing pest infestations [7]. Hence, this newly emerging but not yet fully understood role of endophytes provides opportunities for anthracnose management. Compared to non-volatile metabolites, endophytic VOCs have significant advantages in biological control. Due to their chemical proprieties, VOCs can spread over large distances, thus having a long-distance range of action, and are easily degraded [5].

Several studies have investigated the fungal endophytic communities of olive tree tissues using cultivation-dependent [8,9,10,11] and independent methods [12,13]. Curiously, some of these studies have shown that fungal endophytes colonizing an olive tree may play an important role in protecting the host plant against anthracnose [10,11]. Indeed, olive tree cultivars with different susceptibilities to anthracnose were shown to harbor distinct endophytic fungal communities under the same pathogen pressure [11]. Each olive cultivar apparently was selective towards specific endophytic taxa, which may confer these differences in host susceptibilities towards anthracnose. The differences found in fungal ecological function profiles among olive tree cultivars, with some cultivars showing a high rate of beneficial endophytes than others, reinforce our hypothesis [12]. Some of the beneficial endophytes associated with olive trees belong to fungal genera with a recognized role in controlling plant pathogens. In this regard, both the *Aureobasidium* and *Sarocladium* genera hold great promise in the protection of olive trees against diseases, due to their recognized ability to act as biocontrol agents against an array of plant pathogens [14,15,16,17,18,19,20].

Thus, the main aim of this study was to evaluate the ability of the endophytes *A. pullulans* and *S. summerbellii*, previously isolated from an olive tree cultivar moderately resistant to anthracnose, to inhibit *C. acutatum* growth and anthracnose symptoms. An important prerequisite for developing and applying effective microbial control agents is a solid knowledge of their mode of action. Therefore, the involvement of VOCs in enhancing protection towards anthracnose provided by the most promising biocontrol endophyte was also studied. This work is expected to identify endophytes and volatile compounds that can be used as starting points for developing new products in the management of olive anthracnose.

## 2. Materials and Methods

### 2.1. Microbial Isolates and Inocula Production

The endophytes *Aureobasidium pullulans* (strain CIMO 19DM275) and *Sarocladium summerbellii* (strain CIMO 19DM011) were previously isolated from symptomless leaves of an olive tree, as detailed in [21,22], and kept in the microbial collection of the Mountain Research Center (CIMO-CC) in 30% (*v*/*v*) glycerol solution at −80 °C. Briefly, these endophytes were isolated by inoculating surface-sterilized leaf fragments previously dissected into small segments (ca. 4–5 mm) on potato dextrose agar (PDA) medium [21,22]. Their identification was based on sequencing of the internal transcribed spacer (ITS) of ribosomal DNA (ITS1, 5.8S rRNA gene and ITS2) [21,22]. The pathogenic fungus *Colletotrichum acutatum* (strain CIMO 15FM003) was obtained from CIMO-CC. This fungus, which is one of the main causal agents of olive anthracnose, was previously isolated from the inner tissues of naturally infected olives and identified by sequencing the ITS region of rDNA using both the universal ITS1 and ITS4 primers [23], and degenerate primers Coll1F and Coll3Rb [24].

The microbial inocula used in the experiments were prepared from frozen stocks by transferring yeast cells or fungal spores to PDA medium. The microorganisms were grown at room temperature for 5 (for yeast) or 15 (for filamentous fungi) days, and yeast or spores produced were then scraped from the agar plates with a sterile rod and suspended in 30 mL of sterile 0.025% (*v*/*v*) Tween 80. The concentration of both yeast cells and fungal spores was adjusted to 1 × 10^6^ cells or conidia/mL with sterile 0.025% (*v*/*v*) Tween 80, in a Neubauer hemocytometer, under a light microscope (Leica DM500), and further used as inocula.

### 2.2. Antagonist Effect of Endophytes against C. acutatum

The antagonistic activity of the endophytes *A. pullulans* and *S. summerbellii* against the causal agent of anthracnose, *C. acutatum*, was evaluated by using in vitro (dual culture) and in vivo (detached fruit) assays. In both assays, the antifungal activity of endophytes was assessed by measuring the inhibition rates in mycelial growth or disease development, sporulation and spore germination of *C. acutatum*.

#### 2.2.1. In Vitro Assays

Dual cultures between the endophytes and *C. acutatum* were established on PDA medium containing 1% (*w/v*) of fresh olive pulp. This medium was prepared by mixing previously ground olives of cv. *Cobrançosa* with a maturation index of 3 (epidermis is red or purple in more than half of the fruit), which was determined according to the IOC guidelines [25], before autoclaving at 121  °C for 15  min. Petri dishes (9 cm diameter) containing 10 mL of this medium were inoculated with 10 µL of cell/spore suspension (10^6^ cells or spores/mL) of each fungus, on opposite sides of the plate (about 3 cm apart). As a control, single *C. acutatum* cultures were prepared. Five replicates of each combination were performed, and the plates were sealed with parafilm and incubated in the dark at 25 ± 2 °C. The radial growth of the pathogen towards the interacting endophyte was evaluated over ten days and used to estimate the inhibition (%) of growth of *C. acutatum* by the endophyte using the formula: Inhibition (%) = [(C − T)/C] × 100, where C and T are the radius (mm) of the *C. acutatum* colony in the control and dual culture plates, respectively. At the end of the assay, the number of spores produced by the pathogen and their viability were also assessed. For sporulation assessment, an agar plug from the interaction zone was transferred into a tube with 15 mL of sterile 0.025% (*v*/*v*) Tween 80. After vortexing for 1–2 min, the concentration of *C. acutatum* spores in the suspension was estimated in a Neubauer counting chamber, and results were expressed in spores/mL. To test spore germination, glass microscope slides containing water agar (15 g/L agar-agar) were inoculated with the same spore suspension used to quantify sporulation. After incubation, at 25 ± 2 °C in the dark for 12 h, the germination percentage was evaluated by counting the number of germinated and non-germinated spores, from a total of 300 spores per glass microscopic slide. 

#### 2.2.2. In Vivo Assays

For the establishment of fruit bioassays, symptomless olive fruits from cv. *Cobrançosa* were used with a maturation index of 3 [25]. After washing in running water, the olives were surface-sterilized through sequential immersion in 70% (*v*/*v*) ethanol for 1 min, and 3–5% (*v*/*v*) sodium hypochlorite for 2 min, and then rinsed three times (1 min each) with sterile distilled water. Fruits were then air-dried and placed on round glass flasks (9 cm in diameter and 10 cm in height) containing sterilized filter paper (Whatman grade 4). Each flask had five olives that were inoculated by distributing 1 mL of cell/spore suspensions of the endophyte (10^6^ cells or spores/mL) over them. After 3 days, they were additionally inoculated with 1 mL of *C. acutatum* spore suspension (10^6^ spores/mL). Controls were performed by inoculating olives with 1 mL of sterile aqueous solution of 0.025% (*v*/*v*) Tween 80, or endophyte/pathogen spore suspension (10^6^ spores/mL). Each olive treatment was performed with 5 replications. Inoculated and control fruits were incubated at 25 ± 2 °C, under a daylight regime, and the filter paper was kept wet during the experiment to maintain the high humidity necessary for infection. Both disease incidence (i.e., percentage of infected fruits) and disease severity (i.e., the proportion of fruit area that is affected) were assessed every 3 days, for 21 days after pathogen inoculation. Disease incidence (%) was determined as the number of infected fruits divided by the total number of tested fruits, multiplied by 100. Disease severity was determined by using a 0 to 5 rating scale, where 0 = no visible symptoms, 1 = visible symptoms affecting <25% of the fruit surface, 2 = 25–50%, 3 = 50–75%, 4 = 75–100%, and 5 = fruit completely rotted with abundant conidia in a gelatinous matrix (soapy fruit) [26]. The area under the disease progress curve for disease incidence (AUDPCi) and severity (AUDPCs) was further calculated for each replication following the procedure described by [26]. Briefly, AUDPC was calculated using the following formula:

$${\mathrm{AUDPC}}_{}=\overset{n}{\underset{i=1}{\sum }}[(I_{i+1}+I_{i})/2]\left(t_{i+1}-t_{1}\right)$$

where (I) is the incidence (%) or severity (%) at the ith observation, ti is the time (days) at the ith observation and n is the total number of observations. At the end of the assay, the sporulation and viability of the pathogen in inoculated olives with pathogen or endophyte + pathogen were evaluated. For this, a spore suspension was obtained by transferring an olive into a tube with 15 mL of sterile 0.025% (*v*/*v*) Tween 80. After vortexing for 1–2 min, the concentration and viability of *C. acutatum* spores were evaluated using the same procedure described above for dual culture assays. 

### 2.3. Characterization of Volatile Compounds Emitted during Fungal Interaction

Among the endophytes studied, *A. pullulans* showed the greatest antagonistic activity against *C. acutatum* in either in vitro or in vivo assays (please see Results). Thus, the ability of this endophyte to produce VOCs with antimicrobial activity was further studied, aiming to elucidate its mechanisms of action against *C. acutatum*. Accordingly, a bioassay with olives was established following a similar procedure previously described. Briefly, five previously sterilized healthy olives of cv. *Cobrançosa* (with a maturation index of 3) were introduced in 50 mL flasks (Duran Gaines Synth, Bioblock), inoculated with 1 mL of cell suspension of *A. pullulans* (10^6^ cells/mL) and, after 3 days, additionally inoculated with 1 mL of *C. acutatum* spore suspension (10^6^ spores/mL). Controls were performed by inoculating olives with 1 mL of sterile solution of 0.025% (*v*/*v*) Tween 80 (mock inoculation), with the endophyte (1 mL at 10^6^ cells/mL) or with the pathogen (1 mL at 10^6^ spores/mL). After inoculation, the flasks were immediately sealed with a polypropylene cap with polytetrafluoroethylene/silicon septum (Duran) and incubated at 25 °C ± 2 °C, under a daylight regime. Five replicates for each treatment were performed.

Based on the results obtained in the in vivo bioassays (please see Results), volatile sampling took place 2, 8 and 16 days after pathogen inoculation. At each sampling time, the volatiles were analyzed by headspace solid phase microextraction gas chromatography coupled to mass spectrometry (HS-SPME-GC/MS), following a similar procedure used by [27]. The vial was placed in a water bath at 30 °C for 5 min to release volatile compounds. Then, under the same conditions of temperature and agitation, the SPME fiber (divinylbenzene/carbonex/polydimethylsiloxane) (DVB/CAR/PDMS 50/30 μm) (Supelco, Bellefonte, PA, USA) was exposed for 30 min for adsorption of the volatile compounds in the headspace. Volatile compounds were removed from the fiber by thermal desorption (220 °C) for 1 min in the chromatograph injection port. The fiber was kept in the injection port for 10 min for cleaning and conditioning for further analysis. The gas chromatograph used was a Shimadzu GC-2010 Plus equipped with a Shimadzu GC/MS-QP2010 SE mass spectrometer detector. A TRB-5MS column (30 m × 0.25 mm × 0.25 μm) (Teknokroma, Spain) was used. The injector was set at a temperature of 220 °C, and the manual injection was performed in splitless mode. The mobile phase consisted of helium 5.0 (Linde, Portugal), at a linear velocity of 30 cm/s and a 24.4 mL/min flow rate. The oven temperature was 40 °C for 1 min, followed by an increase of 2 °C/min until reaching 220 °C. The ionization source was maintained at 250 °C with an energy of 70 eV and a current of 0.1 kV. All mass spectra were obtained by electronic ionization in the m/z range 35–500. Compounds were identified by comparing the mass spectra and through the Kovats index using databases such as NIST 69, PubChem and ChemSpider. Retention indices were obtained using a commercial n-alkane series, C7-C30 (Sigma-Aldrich, St. Louis, MS, USA), by direct splitless liquid injection (1 μL), while all further conditions of GC and MS were settled for the volatile analysis. Retention indices were calculated according to the Kovats index. The identified volatile compounds were expressed based on the areas determined by TIC (total ion chromatogram) integration.

### 2.4. Data analysis

The results of the in vitro and in vivo assays and volatile compounds are presented as the mean of each parameter accompanied by the respective standard deviation (SD). To determine differences among the means, one-way analysis of variance (ANOVA) with PAST v4.03 software was conducted, and the averages were compared using Tukey’s test (*p* < 0.05). Before analysis, the normality of data was checked by using the Shapiro–Wilk test. The same software was used to generate the heatmap, box plot and bar charts. 

To analyze the effect of the different treatments (i.e., olives inoculated with *C. acutatum*, *A. pullulans*, *A. pullulans* + *C. acutatum* or Tween) and time of fungi interaction (2, 8 and 16 days) on the volatile composition, permutational multivariate analysis of variance (PERMANOVA) was performed by using the function *adonis2* from the package “vegan” [28] in R software v.3.5.1 205 [29]. The volatile compounds differently emitted in olives inoculated with fungi (*C. acutatum*, *A. pullulans*, *A. pullulans* + *C. acutatum*) relative to olives inoculated with Tween (control) were estimated by calculating the log2-fold changes. The same approach was applied to calculate the log2-fold change for all the sampling times of volatile compounds (i.e., 2, 8 and 18 days).

Principal component analysis (PCA) was performed to identify the volatile compounds that best discriminate the different treatments over the interaction of fungi. This analysis was performed in R software v.3.5.1 205 [29] using the function *pca* from the “FactoMineR” package [30]. Next, the biplot of the two first PCs was drawn using the *fviz_pca_biplot* function from the “factoextra” package [31]. PCA arrows represent the contribution of each volatile compound to the two components (length of the arrow), and the specific gradient color denotes their contribution to the explanation of the greatest variance in the dataset.

## 3. Results

### 3.1. In Vitro Interaction between Endophytes and C. acutatum

**Figure 1:**
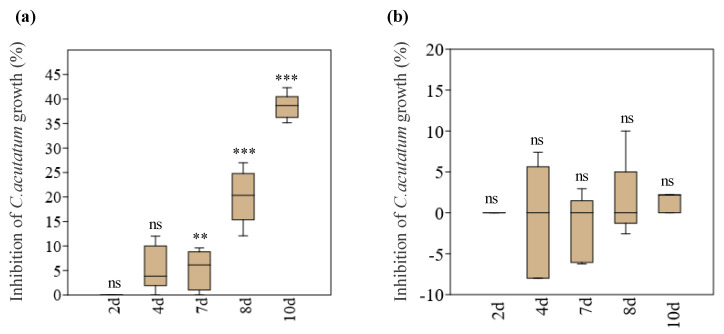
Inhibition (%) of *Colletotrichum acutatum* growth by the endophytes *Aureobasidium pullulans* (**a**) and *Sarocladium summerbellii* (**b**), after 2, 4, 7, 8 and 10 days of interaction. Box plots depict medians (central horizontal lines), inter-quartile ranges (boxes) and 95% confidence intervals (whiskers). Asterisks indicate statistical significance compared with the control (*n* = 5), at *** *p* < 0.001 and ** *p* < 0.01, within each day of interaction. ns: not significant.

Dual cultures between *A. pullulans* and *C. acutatum* and *S. summerbellii* and *C. acutatum* and the control (single cultures of *C. acutatum*) were established on PDA amended with 1% (*w/v*) of macerated fresh olives. Among the two endophytes tested, only *A. pullulans* showed the capacity to significantly inhibit the growth of *C. acutatum* (Figure 1). The inhibitory effect of *A. pullulans* on the growth of *C. acutatum* was obvious after 7 days of interaction, by significantly decreasing (*p* < 0.01) the pathogen’s growth by 5% on average in relation to the control, and reaching the highest growth reduction (on average 39%) at 10 days of interaction, the moment after which the colonies of both microorganisms establish contact (Figure 1a). In contrast, *S. summerbellii* did not significantly change the pathogen’s growth when compared to the control (Figure 1b).

**Figure 2:**
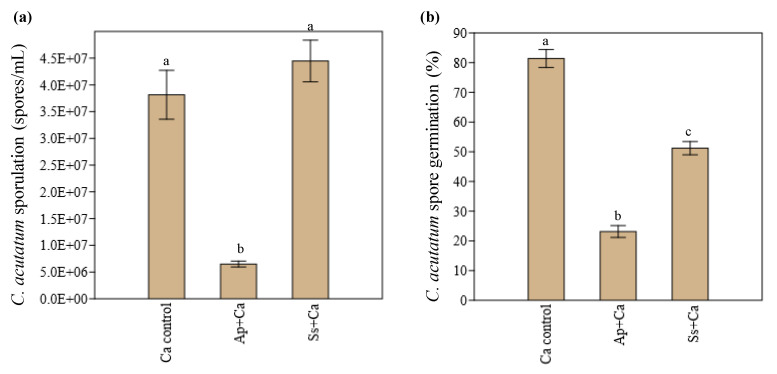
Sporulation (**a**) and spore germination (**b**) of *Colletotrichum acutatum* in co-culture with *Aureobasidium pullulans* (Ap + Ca) or *Sarocladium summerbellii* (Ss + Ca) and in control (single culture of *C. acutatum*, Ca control), after 10 days of interaction. Bars with different lowercase letters indicate significant differences (*p* < 0.05, *n* = 5).

Marked differences were also observed among the two endophytes tested on their capacity to inhibit both the sporulation and germination of *C. acutatum* (Figure 2). *A. pullulans* significantly (*p* < 0.001) reduced the sporulation (up to 5.9-fold) and germination (up to 3.5-fold) of *C. acutatum*, in relation to the control (*C. acutatum* single culture), while *S. summerbellii* only showed the capacity to significantly (*p* < 0.001) inhibit the germination of *C. acutatum* (up to 1.6-fold in relation to the control).

### 3.2. In Vivo Interaction between Endophytes and C. acutatum

**Figure 3:**
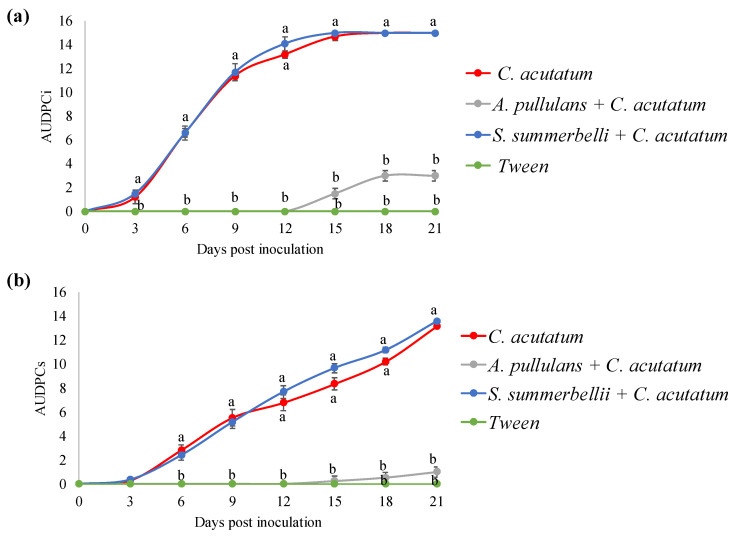
Area under the disease progress curve of incidence (AUDPCi; (**a**)) and severity (AUDPCs; (**b**)) of anthracnose in olive fruits for the different treatments, over 21 days of post-inoculation with the pathogen *Colletotrichum acutatum*. Treatments: fruits inoculated with Tween 80 (mock-inoculated), with the pathogen (control) and with the endophyte and, 3 days later, with the pathogen (*A. pullulans* + *C. acutatum* or *S. summerbellii* + *C. acutatum*). For each day, mean values of the several treatments followed by different letters are significantly different (*p* < 0.05, *n* = 5).

The capacity of both endophytes *A. pullulans* and *S. summerbellii* to reduce the incidence (AUDPCi) and severity (AUDPCs) of anthracnose, caused by the pathogen *C. acutatum*, was evaluated by artificial inoculation of detached olives. The results show that *A. pullulans* significantly reduced (*p* < 0.001) the progress curve for the incidence (up to 10-fold) and severity (up to 35-fold) of anthracnose in comparison with olives inoculated solely with *C. acutatum* (Figure 3). On day 15, 100% incidence was reached in olives inoculated solely with *C. acutatum*, while in the *A. pullulans* + *C. acutatum* treatment, the incidence was 20% and remained practically stable until the end of the bioassay. Similarly, olives inoculated with *A. pullulans* and *C. acutatum* did not exceed the severity index of 2 (25–50% of the infected area) until the end of the bioassay, while olives inoculated with *C. acutatum* reached the severity index of 5 by the end of the bioassay. An interesting delay by 15 days in the appearance of disease symptoms was also observed in the presence of *A. pullulans* as opposed to 3 days in the *C. acutatum* control.

The endophyte *S. summerbelli* did not reduce the disease progression rate compared to the control (i.e., olives inoculated solely with *C. acutatum*), either for incidence (*p* = 0.766) or severity (*p* = 0.371). Indeed, both the incidence and severity of anthracnose in olives inoculated with *S. summerbelli* + *C. acutatum* increased steadily throughout the 21 days of olive fruit monitoring and at the same rate as that in olives inoculated with *C. acutatum* (Figure 3). In both treatments, 100% incidence and a severity index of 5 were reached by days 15 and 21, respectively, with characteristic anthracnose symptoms (i.e., mass of orange conidia) starting to show from the ninth day.

**Figure 4:**
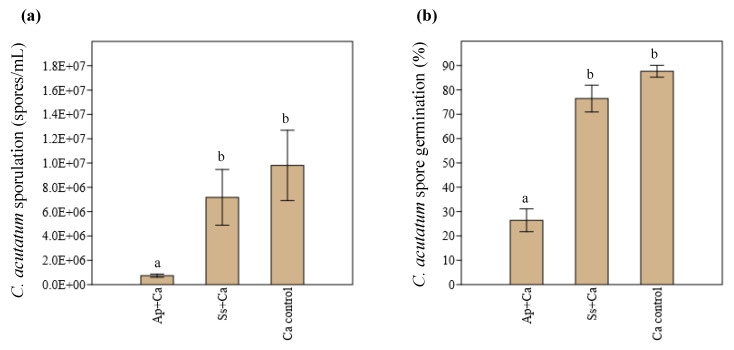
Sporulation (**a**) and spore germination (**b**) of *Colletotrichum acutatum* in the presence of the endophytes *Aureobasidium pullulans* (Ap + Ca) and *Sarocladium summerbellii* (Ss + Ca) and in control (*C. acutatum*—Ca control), in olive bioassays, after 21 days of pathogen inoculation. Bars with different lowercase letters indicate significant differences (*p* < 0.05, *n* = 5).

At the end of the olive bioassay, both the sporulation and germination of *C. acutatum* in the presence of *A. pullulans* and *S. summerbellii* were evaluated and compared to those of the control (olives solely inoculated with *C. acutatum*). The results show great differences among the two endophytes in their capacity to inhibit both the sporulation and germination of *C. acutatum* in olives (Figure 4). *A. pullulans* significantly (*p* < 0.05) reduced the sporulation and germination of *C. acutatum*, by 90% and 70%, respectively, in relation to the control. In contrast, no significant difference in the sporulation (*p* = 0.497) and germination (*p* = 0.099) of *C. acutatum* was observed among the *S. summerbellii* + *C. acutatum* treatment and control.

### 3.3. Volatile Compounds Produced during the Interaction of A. pullulans–C. acutatum

Among the two endophytes tested, *A. pullulans* showed the greatest biocontrol potential against *C. acutatum*. The results from the dual culture of *A. pullulans*–*C. acutatum* show that the endophyte inhibits the growth of the pathogen before mycelial contact and that none of the isolates were able to overgrow the other (data not shown). Thus, it is likely that this inhibitory effect was due to diffusible and/or volatile compounds with antimicrobial activity produced by *A. pullulans*. Therefore, during the in vivo interaction between *A. pullulans* and *C. acutatum*, the production of VOCs after 2, 8 and 16 days of olive inoculation with the pathogen was studied. These sampling times were selected based on results from the bioassay that showed changes in the inhibitory effect of *A. pullulans* over anthracnose progression with time: 2 days (no inhibition), 8 days (moderate inhibition with statistical significance) and 16 days (maximal inhibition). Overall, a total of 38 compounds were detected, 37 of which were putatively identified by comparison to the NIST library of mass spectra (Table 1). The identified compounds belong to seven different chemical classes (Table S1). Alcohols, aldehydes and ketones were the most diversified chemical classes, with 14, 8 and 7 different compounds, respectively (Table S1), while the most abundant ones were alcohols followed by alkanes, accounting for 62% and 17% of the total VOC abundance, respectively (Figure S1).

The emitted VOCs by the inoculated olives were qualitatively and semi-quantitatively different among the four treatments (Table 1; Figure S2a), with the type of treatment accounting for 13% of the total VOC variance (PERMANOVA, *p* < 0.001; Table S2). Overall, olives inoculated with *C. acutatum* or Tween showed the highest number of volatiles (30 and 33, respectively). By contrast, the total number of VOCs produced in olives inoculated with *A. pullulans* or *A. pullulans* + *C. acutatum* was lower (25 and 26, respectively). Around 53% of the detected compounds were common to all four treatments, with five VOCs exclusively identified in olives inoculated with Tween and one volatile (2-undecanone) in olives inoculated with *C. acutatum*. Curiously, four VOCs (2-hexanone, 2-heptanone, 2-octanone and limonene) were exclusively identified in olives inoculated with *A. pullulans* + *C. acutatum* or *C. acutatum*. 1-Hexanol was the most abundant compound in olives inoculated with *A. pullulans*, while in the other treatments, it was 2-methyl-1-butanol. In comparison to the control (i.e., olives inoculated with Tween), both *A. pullulans* + *C. acutatum* and *C. acutatum* showed greater changes in the volatile profile than *A. pullulans* (Figure S3). Overall, the abundance of most VOCs emitted in the control (around 53%) was significantly reduced (up to *p* < 0.05) in fungal-inoculated olives, with only 16% increasing in abundance. In particular, 1-heptanol, 2-hexenal and methoxy-phenyl-oxime were the most depleted in olives inoculated with *A. pullulans*, *A. pullulans* + *C. acutatum* and *C. acutatum*, respectively, while Z-3-hexen-1-ol (in *A. pullulans*) and 2,2,4-trimethyl-pentane (in both *A. pullulans* + *C. acutatum* and *C. acutatum*) were the most enhanced.

Similarly, differences were found in VOC production over the pathogen–endophyte interaction, with the sampling time accounting for 42% of the total variance in the VOCs emitted (PERMANOVA *p* < 0.001; Table S2). The results show a decrease in the number of VOCs produced throughout the experiment, from 30 VOCs on day 2, to 28 VOCs on day 8 and 19 VOCs at the end of the assay (Table 1; Figure S2b). Some VOCs were detected at a specific time point. Overall, a total of eight VOCs were exclusively detected on day 2 and, afterwards, reduced to four (on day 8) and two (on day 16). The main VOC emissions also varied over time, with hexanal, 2,2,4-trimethyl-pentane and 2-methyl-1-butanol being the most abundant on days 2, 8 and 16, respectively. Differences in the individual VOC amount between olives inoculated with Tween (control) and fungi were noticed mostly on days 2 and 8 of the experiment (Figure S4). At these two sampling times, the abundance of 63% of the total VOCs emitted in fungal-inoculated olives decreased significantly (at least *p* < 0.05) when compared to the control. In contrast, at the later stages of the assay (day 16), the fold change in the VOC amount between olives inoculated with fungi and the control was negligible, with only a few VOCs increasing their amount in the presence of fungi.

**Figure 5:**
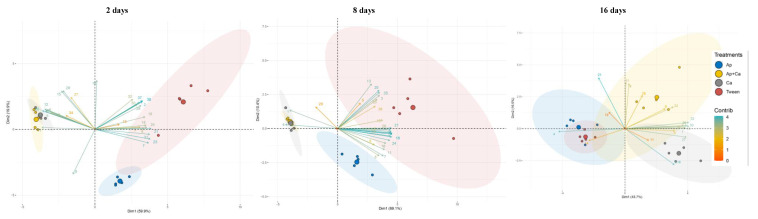
Principal component analysis score plots obtained from the volatile profile of olives inoculated with *Aureobasidium pullulans* and *Colletotrichum acutatum* (Ap + Ca), or solely with *A. pullulans* (Ap), *C. acutatum* (Ca) or Tween, after 2, 8 and 16 days of pathogen inoculation. Each number corresponds to a particular volatile compound, as indicated in Table 1. The gradient color represents the contribution of each variable (i.e., volatile compounds) to the explanation of the greatest variance in the dataset. Each circle represents treatments, with the ones with the greater size representing the average.

To identify which VOCs are characteristic of each treatment, principal component analysis (PCA) was performed with VOCs analyzed after 2, 8 and 16 days of pathogen inoculation (Figure 5). The PCA showed a high variance, around 80%, 80% and 60% for days 2, 8 and 16, respectively, considering the two principal components (PC1 and PC2). For day 2, the PCA grouped the *C. acutatum* (Ca) and *A. pullulans* + *C. acutatum* (Ap + Ca) treatments together, which were characterized by the VOCs 2-methyl-1-propanol, 2-methyl-1-butanol, 2-hexanone, 1-hexanol, 2-heptanone and limonene. These two treatments were separated from the *A. pullulans* (Ap) and Tween treatments, which correlated positively with an array of VOCs, with hexanal, benzeneacetaldehyde, [1S-(1α,2α.,5β)]-cyclohexanol and 5-methyl-2-(1-methylethyl) being the most discriminant. The PCA for day 8 showed a similar trend, with treatments Ca and Ap + Ca being clearly separated from the other treatments due to the production of 2-methyl-1-propanol and 2,2,4-trimethyl-pentane. On day 16, this pattern changed, with a separation between the Ca, Ap + Ca and Ap/Tween treatments being noticed based on the volatiles produced. On this day, the most characteristic VOCs of the Ca treatment were 6-methyl-5-hepten-2-one and 2-nonanone, while the Ap + Ca treatment was positively correlated with an array of compounds, particularly Z-3-hexen-1-ol, benzyl alcohol and nonanal. The Ap and Tween treatments were positively related to the production of 2,2,4-trimethyl-pentane.

## 4. Discussion

One of the major challenges in developing microbial biological control agents against pathogens is related to the method used for their screening [32]. The selection of antagonists often relies on in vitro dual culture assays in an artificial medium; however, the antagonism observed in this approach is not always reflected in in vivo assays [33]. Indeed, the in vitro screening method is only based on inhibition of pathogen growth due to antimicrobial metabolites [32]. However, other modes of action of the antagonist can contribute in planta to a better protection of plants from damage by diseases [32]. Screening methods for microbial biological control agents may therefore rely on assays that take into account the interaction between the pathogen, host plant and antagonist [32,33]. In our work, the antagonistic effect of *A. pullulans* and *S. summerbelli* against the causal agent of olive anthracnose, *C. acutatum*, was assessed based on this approach by using both in vitro and in vivo assays. In the first assay, dual cultures were established in a medium containing macerated olives, in an attempt to mimic the natural habitat under which antagonists should be active, while in vivo assays were performed in detached fruits. Similar results were obtained in both assays, by showing the greatest efficacy of *A. pullulans* in controlling *C. acutatum*. This endophyte significantly reduced the development of disease, as well as the growth, sporulation and germination of *C. acutatum*. *A. pullulans* is a well-known biocontrol agent against several pathogenic fungi, especially postharvest pathogens, but also pathogens present in the field [14,18,19]. For instance, *A. pullulans* was shown to suppress the mycelium growth and/or conidia germination of *Botrytis cinerea* [18,19], *Alternaria alternata* [19], *Colletotrichum acutatum* [15], *Penicillium expansum*, *Penicillium digitatum* and *Penicillium italicum* [14], under in vitro and/or in vivo conditions. Its potential in the biocontrol of olive anthracnose was previously reported as well. In field trays, this yeast was shown to significantly reduce anthracnose severity by 40% and latent infection by 14% [17]. Despite the inability of *S. summerbellii* to suppress *C. acutatum* in the present study, some endophytic species of the genus *Sarocladium* have already been reported to display significant antifungal activity against a broad spectrum of pathogens, including the genus *Colletotrichum* [16].

The in vitro inhibitory effect displayed by *A. pullulans* against *C. acutatum* in the present study started to occur prior to physical contact among the microbes, suggesting that the inhibition was caused by diffusible and/or volatile compounds produced by the endophyte. Therefore, changes in the VOC emission among olives inoculated with fungi (i.e., *A. pullulans*, *C. acutatum* or *A. pullulans* + *C. acutatum*) and Tween (control) were evaluated after 2, 8 and 16 days post-inoculation. Overall, the VOCs detected were mainly alcohols, aldehydes and ketones, with alcohols being the most abundant and diversified compounds. This result is consistent with previous works that identified these three chemical classes among the most abundant volatile compounds produced by fungi [34]. These classes encompass VOCs with recognized antimicrobial effects against several plant pathogens [14,35].

In the present study, the composition of the VOC blend was revealed to be different among treatments, with 2-undecanone being exclusive to olives inoculated only with *C. acutatum*. This result suggests that this compound may play an important role in the development of anthracnose, probably being required for the pathogenicity of *C. acutatum*, as previously suggested for other pathosystems [36]. Alternatively, this VOC may be produced by olives to prevent *C. acutatum* infection, as plants are known to protect themselves from pathogens by synthesizing diversified metabolites, including VOCs [37]. 2-Undecanone has been previously reported to inhibit the growth of the pathogens *Verticillium dahliae* and *Fusarium oxysporum* [38]; thus, it is likely that it acts as a defense compound produced by olives in response to pathogen challenge. Besides 2-undecanone, 2-hexanone, 2-heptanone, 2-octanone and limonene might also have a similar role in defending plants from *C. acutatum* colonization, due to their exclusive production in olives inoculated with *C. acutatum* or with *A. pullulans* + *C. acutatum*. In this regard, terpenes (that include limonene) constitute one of the largest VOC groups that are assumed to function in plant defense against pathogens [39].

Changes in volatile profiles were also observed over time in the present study, with some VOCs being exclusive to a specific time point, suggesting that microorganisms produce a typical volatile blend depending on their growth and/or interaction stage. We hypothesized that these compounds, which became distinct, may represent signals of the ongoing metabolism derived from the microbial–olive interaction. Accordingly, it is possible that the high variety of VOCs released in the first two days of the assays may be linked to great changes in the metabolism of interacting fungi and olives. Interesting changes in the overall VOC profiles included the dominance of hexanal, 2,2,4-trimethyl-pentane and 2-methyl-1-butanol on days 2, 8 and 16, respectively. These compounds, particularly the aldehyde hexanal and the alcohol 2-methyl-1-butanol, were previously shown to have antifungal proprieties against several postharvest pathogens in fruits and vegetables [14,40,41]. To our knowledge, the antifungal potential of 2,2,4-trimethyl-pentane has not yet been analyzed; however, it belongs to the alkane class that is recognized to comprise compounds with the ability to suppress plant pathogens [42].

Multivariate analysis of our results showed a strong relationship among the volatile profiles between the *A. pullulans* + *C. acutatum* (Ap + Ca) and *C. acutatum* treatments, and their clear separation from the *A. pullulans* treatment, for the first 8 days of the assay. This suggests a greater contribution of *C. acutatum* when compared to *A. pullulans* for the VOC blend composition in the Ap + Ca treatment. In addition, the inoculation of olives with Ap + Ca or *C. acutatum* induced stronger changes in the VOC abundance in relation to the control (olives inoculated with Tween) than the inoculation with *A. pullulans*. Thus, it is likely that metabolic pathways were more changed by the inoculation with Ap + Ca or *C. acutatum* compared to the inoculation with *A. pullulans* alone. The VOCs most positively associated with the Ap + Ca and *C. acutatum* treatments in the first 8 days of the assay included alcohols (2-methyl-1-propanol, 2-methyl-1-butanol, 1-hexanol), ketones (2-hexanone, 2-heptanone), terpenes (limonene) and alkanes (2,2,4-trimethyl-pentane), with most of them previously being reported to have antimicrobial activity against a variety of pathogens [5,14,39]. Curiously, some of these VOCs, particularly 2-methyl-1-propanol and 2-methyl-1-butanol, were previously identified from *A. pullulans* and also showed inhibitory activities against an array of pathogens, including *C. acutatum* [14,19]. The aldehydes hexanal and benzeneacetaldehyde and the alcohol [1S-(1α,2α.,5β)]-cyclohexanol, 5-methyl-2-(1-methylethyl) were the VOCs that most discriminated *A. pullulans* from the other treatments in the present study. The first two molecules are well-known antimicrobials [40,41], and the last one is a bioactive compound that has been found in several medicinal plant species [43]. At the later stages of the assay (on day 16), a clear discrimination between VOC profiles emitted from the *C. acutatum*, Ap + Ca and *A. pullulans* treatments was observed. As the antagonism of *A. pullulans* against *C. acutatum* was the highest at this stage, we hypothesized that these differences in VOC profiles are important in reducing anthracnose development. In this regard, Z-3-hexen-1-ol, benzyl alcohol and nonanal seem to hold great promise due to their highly positive correlation with the Ap + Ca treatment. Z-3-hexen-1-ol is frequently reported to be produced by plants immediately after mechanical damage of their leaves [44], acting as an attractant of beneficial insects to crops and in triggering the plant–plant communication to prevent further insect attacks [45]. As far as we know, the involvement of this compound in plant–microbe interactions has never been reported before. We hypothesized that this compound can also be used by olives to communicate the presence of pathogen infection and/or to act as signals that cause resistance responses. Therefore, the potential role of this compound in reducing anthracnose in olives requires further investigation. Previous studies have shown the ability of benzyl alcohol to inhibit the growth and conidia germination of *Botrytis cinerea* [46,47]. Some authors have also ascribed the antimicrobial effectiveness displayed by several fungi against pathogens, including *C. acutatum*, to benzyl alcohol [48,49]. Nonanal is an aldehyde whose antimicrobial proprieties have been demonstrated against several pathogenic fungi [50,51]. Besides these three VOCs, other compounds were found positively associated with the Ap + Ca treatment. Thus, it is likely that the reduction in anthracnose incidence and severity observed in this treatment was not caused by one or a few VOCs, but instead by various components of the volatile blend that act synergistically. Curiously, on day 16 post-inoculation, 6-methyl-5-hepten-2-one was found to be positively associated with the *C. acutatum* treatment, and this compound was previously reported to be used by the pathogen *Neofabraea vagabunda* for conidial germination on the surface of apples [52]. 2-Nonanone was another compound highly associated with the Ca treatment and was previously reported as a biomarker for the identification of bacterial pathogens [53].

## 5. Conclusions

This work identified *A. pullulans* as a potential candidate in conferring olive tree protection against anthracnose. This yeast was effective in reducing the development of anthracnose, mycelial growth, sporulation and germination of *C. acutatum* either in in vitro or in vivo assays. Its features were ascribed to the production of volatile organic compounds, with Z-3-hexen-1-ol, benzyl alcohol and nonanal being the most promising inhibitory compounds. However, their effectiveness against *C. acutatum* should be further evaluated under in vitro conditions and in the field. The contribution of other antimicrobial compounds, particularly diffusible compounds, to the inhibitory effect displayed by *A. pullulans* against *C. acutatum* cannot be disregarded and should be further deepened. Our results also suggest that some VOCs positively associated with and differently produced in olives inoculated solely with *C. acutatum* are likely to play a role in the pathogenicity and/or development of anthracnose. These findings may provide opportunities to develop new tools for integrated management of anthracnose disease, through the exploitation of *A. pullulans* or VOCs.

## Figures and Tables

**Table 1:** Volatile profile of olives inoculated with *Aureobasidium pullulans* and *Colletotrichum acutatum* (*A. pullulans* + *C. acutatum*), or solely with *A. pullulans*, *C. acutatum* or Tween (controls), 2, 8 and 16 days after pathogen inoculation. Results are expressed in relative percentage of the total chromatogram area (mean ± standard deviation, *n* = 5).

Nº	Compound	*A. pullulans*			*A. pullulans* + *C. acutatum*			*C. acutatum*			Tween		
		2 Days	8 Days	16 Days	2 Days	8 Days	16 Days	2 Days	8 Days	16 Days	2 Days	8 Days	16 Days
1	2-Methyl-1-propanol	-	-	12.2 ± 4.02	5.8 ± 0.90	7.6 ± 1.98	21.7 ± 1.37	3.4 ± 0.72	7.1 ± 1.60	21.9 ± 0.90	-	-	16.1 ± 2.32
2	3-Methyl-butanal	-	-	-	-	-	-	-	-	-	1.4 ± 0.51	-	-
3	2-Methyl-butanal	3.1 ± 1.31	-	-	-	-	-	2.4 ± 0.49	-	-	4.6 ± 1.51	1.8 ± 0.62	-
4	2,2,4-Trimethyl-pentane	-	15.6 ± 6.81	30.8 ± 9.10	-	57.3 ± 4.68	6.1 ± 1.26	-	58.4 ± 6.90	4.7 ± 1.67	-	-	27.9 ± 4.12
5	2-Methyl-1-butanol	-	19.5 ± 5.74	30.1 ± 3.86	17.6 ± 2.99	18.4 ± 2.95	39.5 ± 2.93	11.4 ± 4.87	18.2 ± 6.26	40.2 ± 3.81	-	25.7 ± 10.07	33.8 ± 3.54
6	2-Hexanone	-	-	-	1.2 ± 0.62	-	-	1.2 ± 0.09	-	-	-	-	-
7	Hexanal	23.9 ± 3.53	4.3 ± 2.32	-	3.4 ± 1.70	-	-	8.7 ± 1.92	-	-	27.0 ± 4.20	4.0 ± 2.51	-
8	2-Hexenal	14.5 ± 4.44	2.0 ± 1.72	-	2.2 ± 1.10	-	-	4.5 ± 1.41	-	-	22.4 ± 12.28	1.9 ± 1.43	-
9	*Z*-3-hexen-1-ol	25.2 ± 2.71	12.2 ± 3.14	4.3 ± 1.43	13.7 ± 2.50	3.2 ± 0.90	5.6 ± 0.98	12.4 ± 1.43	2.9 ± 0.63	5.2 ± 0.72	-	9.7 ± 4.24	2.3 ± 0.94
10	*Z*-2-hexen-1-ol	-	2.5 ± 0.84	-	-	-	-	-	-	-	-	4.1 ± 3.84	-
11	1-Hexanol	8.9 ± 5.96	29.4 ± 6.69	15.0 ± 3.93	23.0 ± 3.71	8.5 ± 2.95	13.9 ± 2.58	20.3 ± 3.76	8.5 ± 2.83	16.9 ± 4.09	-	28.4 ± 2.20	11.2 ± 3.88
12	2-Heptanone	-	-	-	3.2 ± 0.94	-	-	3.9 ± 0.91	-	-	-	-	-
13	Methoxy-phenyl-oxime	-	-	-	-	-	-	-	0.2 ± 0.03	-	1.7 ± 2.21	0.5 ± 0.21	-
14	*Z*-hept-4-enol	-	-	-	-	-	-	-	-	-	-	-	0.3 ± 0.03
15	1-Heptanol	-	0.4 ± 0.06	-	1.7 ± 0.56	0.3 ± 0.11	1.0 ± 0.40	1.9 ± 0.30	0.2 ± 0.02	1.2 ± 0.52	0.9 ± 0.41	0.6 ± 0.22	0.6 ± 0.22
16	6-Methyl-5-hepten-2-one	-	0.3 ± 0.06	-	0.7 ± 0.21	-	-	0.5 ± 0.06	0.2 ± 0.06	0.5 ± 0.12	0.8 ± 0.16	0.4 ± 0.09	-
17	2-Octanone	-	-	-	2.4 ± 1.33	-	-	4.8 ± 1.10	-	-	-	-	-
18	*Z*-3-hexen-1-ol acetate	1.4 ± 0.27	0.6 ± 0.06	-	0.9 ± 0.24	-	-	0.8 ± 0.06	-	-	2.2 ± 0.57	0.8 ± 0.18	-
19	Hexyl ester acetic acid	1.4 ± 0.27	2.4 ± 0.38	0.5 ± 0.11	4.2 ± 1.03	0.4 ± 0.14	0.4 ± 0.12	3.6 ± 0.27	0.3 ± 0.09	0.4 ± 0.20	8.3 ± 1.90	3.5 ± 0.71	0.5 ± 0.06
20	Limonene	-	-	-	4.2 ± 0.71	-	-	3.7 ± 0.16	-	-	-	-	-
21	*Not identified*	1.7 ± 0.35	1.4 ± 0.18	0.7 ± 0.11	-	0.5 ± 0.08	1.0 ± 0.18	-	0.4 ± 0.04	-	3.0 ± 0.76	2.0 ± 0.39	0.7 ± 0.09
22	Benzyl alcohol	-	-	-	-	-	0.5 ± 0.16	-	-	0.3 ± 0.19	-	-	0.3 ± 0.07
23	Benzeneacetaldehyde	0.9 ± 0.22	-	-	-	-	-	-	-	-	1.2 ± 0.16	-	-
24	*E*-2-octenal	1.1 ± 0.21	0.4 ± 0.09	-	-	-	-	0.7 ± 0.04	-	-	1.7 ± 0.35	0.5 ± 0.15	-
25	*E*-2-octen-1-ol	-	-	-	-	-	-	-	-	-	-	0.3 ± 0.10	-
26	1-Octanol	-	0.4 ± 0.13	-	1.0 ± 0.40	0.2 ± 0.06	0.7 ± 0.22	1.2 ± 0.14	0.2 ± 0.03	0.8 ± 0.16	0.6 ± 0.23	0.6 ± 0.21	0.4 ± 0.06
27	2-Nonanone	0.8 ± 0.17	0.5 ± 0.13	0.2 ± 0.06	1.4 ± 0.27	0.2 ± 0.04	0.3 ± 0.07	1.7 ± 0.26	0.2 ± 0.06	0.4 ± 0.11	1.3 ± 0.33	0.8 ± 0.19	0.2 ± 0.03
28	Nonanal	1.4 ± 0.26	0.7 ± 0.10	0.3 ± 0.16	1.0 ± 0.16	0.2 ± 0.10	0.4 ± 0.21	1.4 ± 0.13	0.2 ± 0.04	0.3 ± 0.05	2.5 ± 0.65	1.0 ± 0.36	0.3 ± 0.05
29	Phenylethyl alcohol	-	0.3 ± 0.07	2.4 ± 1.55	-	0.6 ± 0.21	3.5 ± 0.86	-	0.5 ± 0.41	1.9 ± 0.77	-	0.4 ± 0.10	2.4 ± 0.68
30	(1S)-1,7,7-trimethyl-bicyclo [2.2.1]heptan-2-one	2.8 ± 0.50	1.6 ± 0.16	0.5 ± 0.08	2.9 ± 0.61	0.5 ± 0.07	0.7 ± 0.11	1.4 ± 0.13	0.5 ± 0.09	0.7 ± 0.03	4.6 ± 1.04	2.6 ± 0.72	0.5 ± 0.08
31	[1S-(1α,2α.,5β)]-Cyclohexanol, 5-methyl-2-(1-methylethyl)	0.5 ± 0.10	0.3 ± 0.05	0.1 ± 0.04	-	0.1 ± 0.01	0.2 ± 0.04	-	0.1 ± 0.02	-	0.8 ± 0.22	0.5 ± 0.15	-
32	Levomenthol	8.2 ± 1.73	5.5 ± 0.54	2.7 ± 0.86	9.0 ± 1.67	1.9 ± 0.28	4.1 ± 0.75	8.4 ± 0.25	1.9 ± 0.39	4.3 ± 0.41	13.7 ± 3.71	9.1 ± 2.80	2.6 ± 0.34
33	Decanal	-	-	-	-	0.08 ± 0.03	-	-	-	-	-	0.3 ± 0.07	-
34	2-Undecanone	-	-	-	-	-	-	0.6 ± 0.14	-	-	-	-	-
35	α-Cubebene	-	-	-	-	-	-	-	-	-	-	0.2 ± 0.07	-
36	Copaene	-	-	-	-	-	-	-	-	-	0.5 ± 0.25	-	-
37	Tetradecane	-	0.2 ± 0.03	0.2 ± 0.04	-	0.08 ± 0.01	0.2 ± 0.04	-	0.08 ± 0.02	0.3 ± 0.06	0.3 ± 0.09	0.3 ± 0.09	0.2 ± 0.03
38	Longifolene	-	0.2 ± 0.04	-	-	-	-	-	0.1 ± 0.03	-	0.4 ± 0.13	0.4 ± 0.28	-

“-”—not detected.

## Data Availability

The data that support the findings of this study are available from the corresponding author upon reasonable request.

## Supplementary Materials

The following supporting information can be downloaded at: https://www.mdpi.com/article/10.3390/microorganisms10030571/s1, Figure S1: Relative abundance (expressed in chromatographic area) of volatile organic compounds per chemical class identified in olives inoculated with *Aureobasidium pullulans* and *Colletotrichum acutatum* (*A. pullulans* + *C. acutatum*), or solely with *A. pullulans*, *C. acutatum* or Tween (controls); Figure S2: Heatmap visualization of volatile compounds identified in olives (a) inoculated with *Aureobasidium pullulans* and *Colletotrichum acutatum* (Ap + Ca), or solely with *A. pullulans* (Ap), *C. acutatum* (Ca) or Tween (controls) (b), after 2, 8 and 16 days of pathogen inoculation. The color intensity (blue to red) refers to the compound abundance in each sample; Figure S3: Log2-fold changes in the abundance of individual volatile organic compounds in olives inoculated with *Aureobasidium pullulans* and *Colletotrichum acutatum* (Ap + Ca), *A. pullulans* (Ap) or *C. acutatum* (Ca) relative to olives inoculated with Tween (control); Figure S4: Log2-fold changes in the abundance of individual volatile organic compounds in olives after 2, 8 and 16 days of fungi inoculation relative to olives inoculated with Tween (control); Table S1: Volatile compounds detected in this study: retention time (RT). The Kovats retention index (RI), and selected ions used as m/z identifiers (QI) [54]; Table S2: Relative importance of the treatment (olives inoculated with *Colletotrichum acutatum*, *Aureobasidium pullulans* or *A. pullulans* + *C. acutatum*) and time of microbial interaction (2, 8 and 16 days) to the volatile composition, as revealed by permutational multivariate analysis of variance (PERMANOVA).
