# Supplementary material for: Biocontrol Ability and Production of Volatile Organic Compounds as a Potential Mechanism of Action of Olive Endophytes against Colletotrichum acutatum
Source: Microorganisms. 2022 Mar 6;10(3):571. doi: 10.3390/microorganisms10030571 (PMC8954755; doi:10.3390/microorganisms10030571)
Supplement: Supplementary file 1 [file microorganisms-10-00571-s001.zip › microorganisms-1582090-supplementary.pdf]

Supplementary material

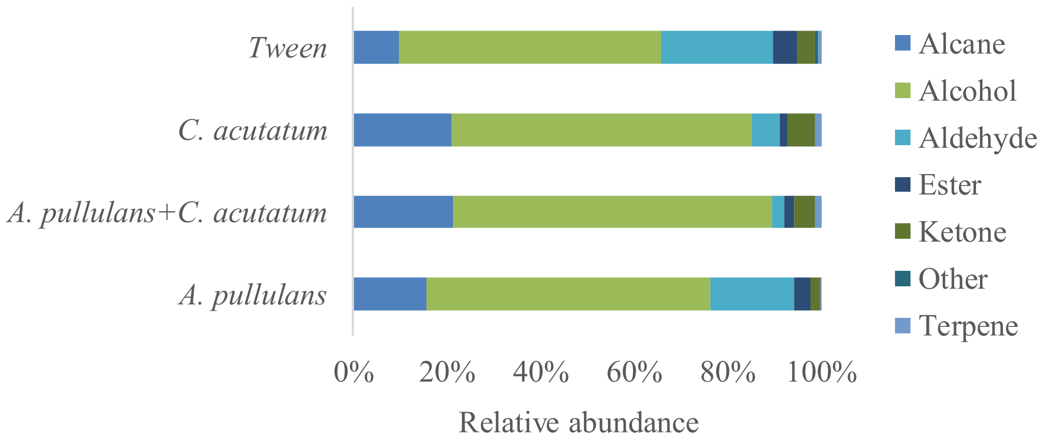

Figure S1. Relative abundance (expressed in chromatographic area) of volatile organic compounds per chemical classes identified in olives inoculated with *Aureobasidium pullulans* and *Colletotrichum acutatum* (*A. pullulans* + *C. acutatum*), or inoculated solely with *A. pullulans*, *C. acutatum* or tween (controls).

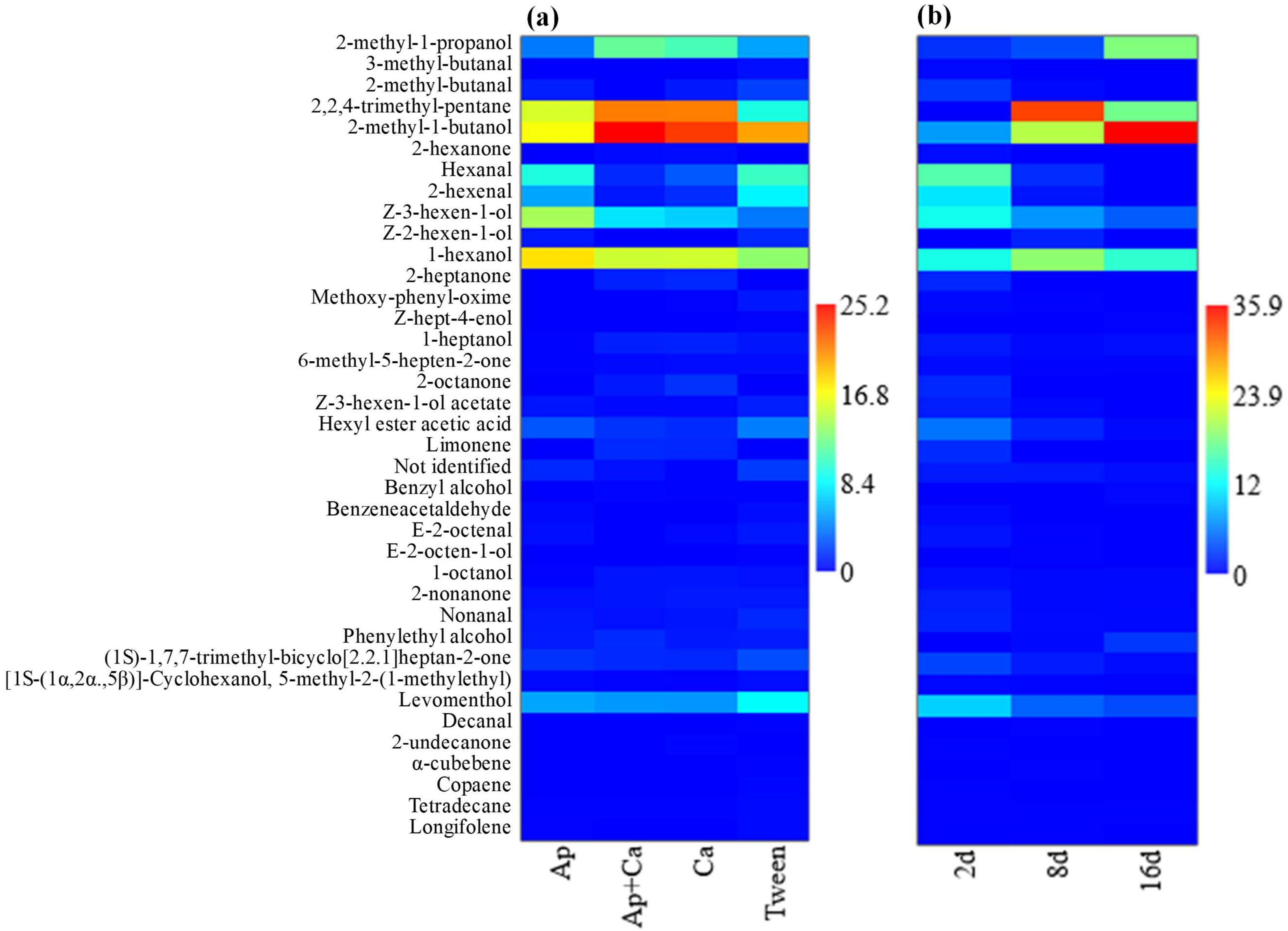

Figure S2. Heatmap visualization of volatile compounds identified in olives (**a**) inoculated with *Aureobasidium pullulans* and *Colletotrichum acutatum* (Ap + Ca), or inoculated solely with *A. pullulans* (Ap), *C. acutatum* (Ca) or tween (controls), (**b**) after 2-, 8- and 16 days of pathogen inoculation. The color intensity (blue to red) refers to compound abundance in each sample.

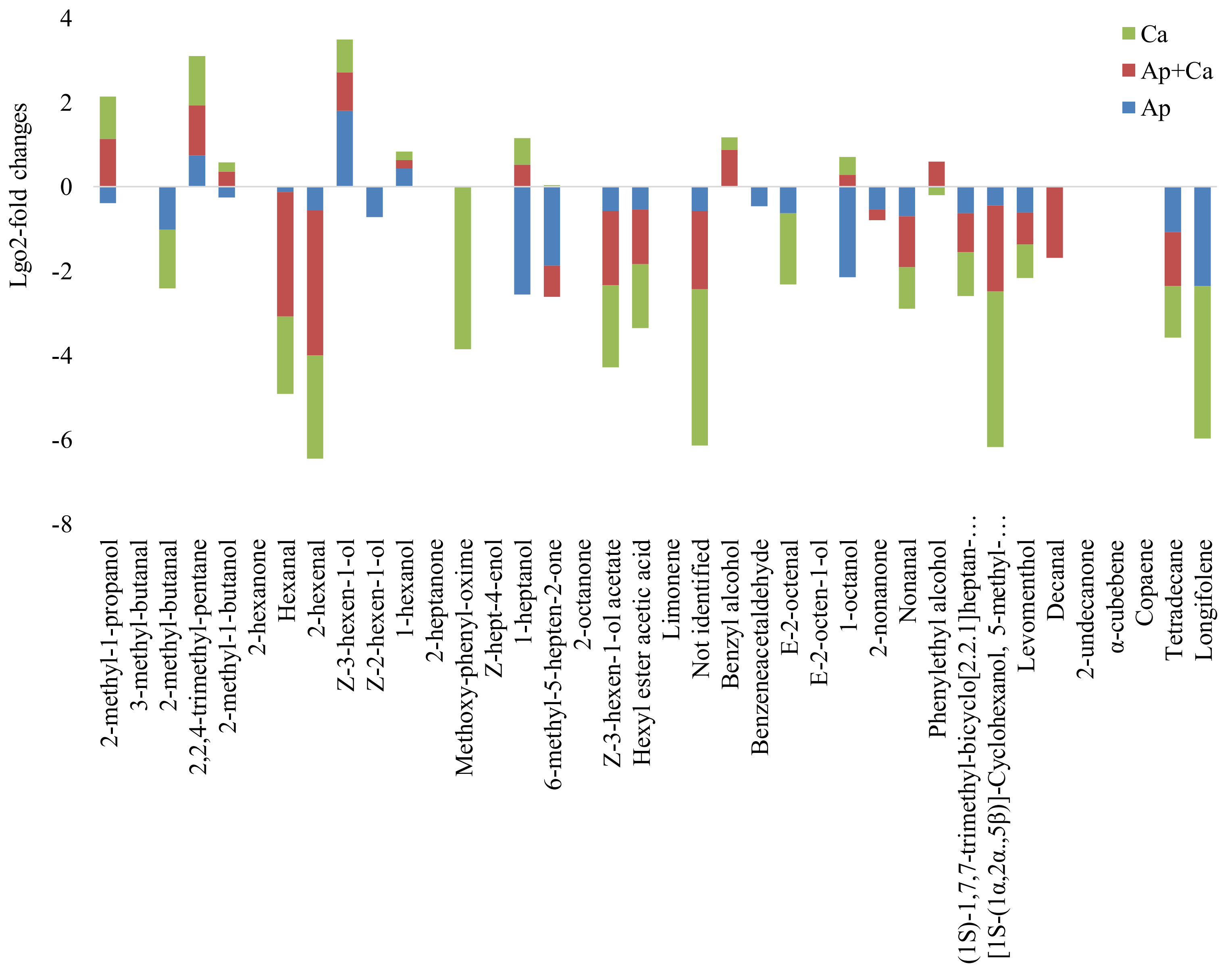

Figure S3. Log2-fold changes in the abundance of individual volatile organic compounds in olives inoculated with *Aureobasidium pullulans* and *Colletotrichum acutatum* (Ap + Ca), *A. pullulans* (Ap) or *C. acutatum* (Ca) relative to olives inoculated with tween (control).

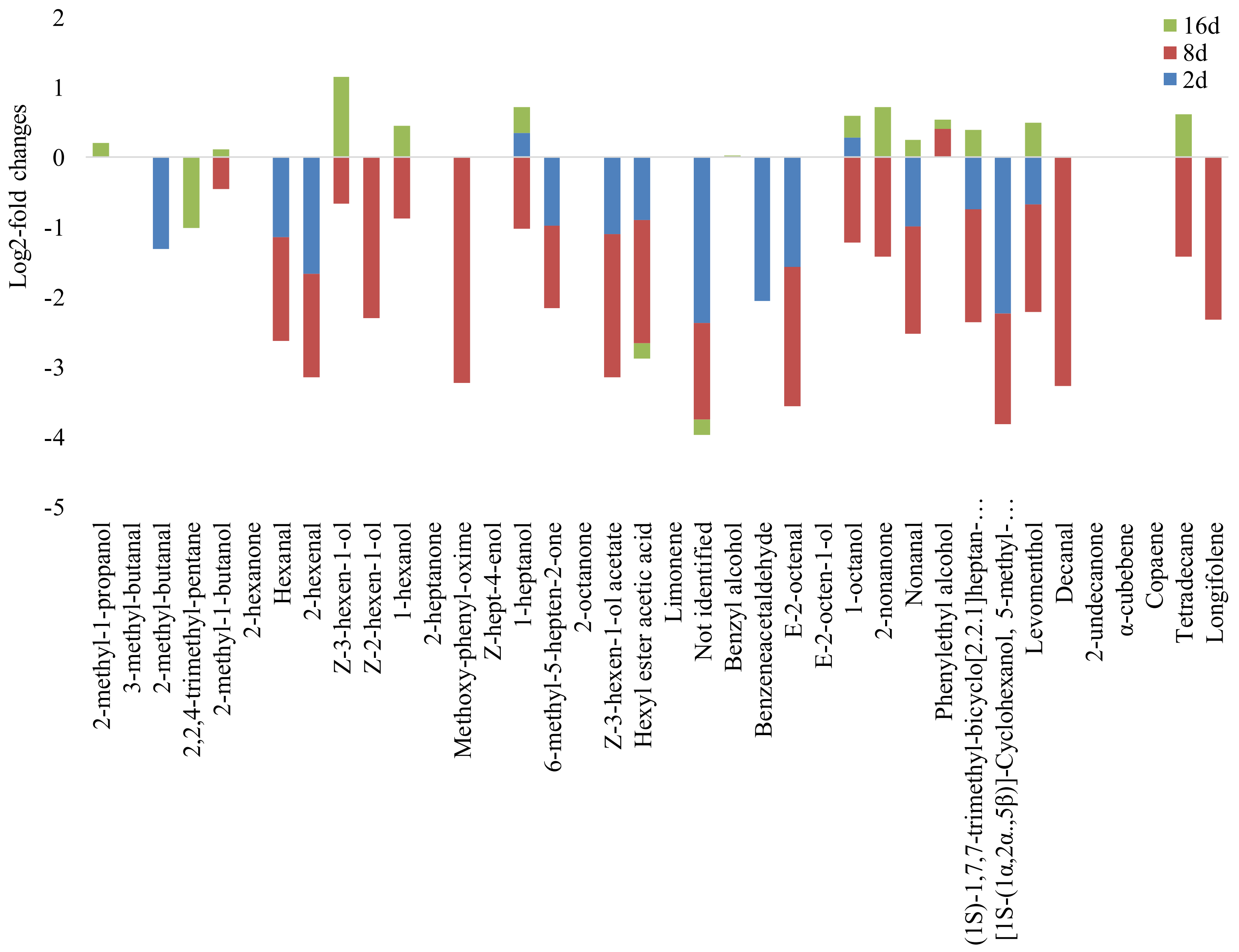

Figure S4. Log2-fold changes in the abundance of individual volatile organic in olives after 2-, 8- and 16 days of fungi inoculation relative to olives inoculated with tween (control).

Table S1. Volatile compounds detected in this study: retention time (RT). Kovat's retention indices (RI), and selected ions used as m/z identifiers (QI).

RT (min)	RIcal[a]	RIlit[b]	Compounds	QI (m/z)
			**Alkane**	
4.534	690	689	2,2,4-trimethyl-pentane	56/57/99
46.824	1298	1400	Tetradecane	43/57/71
			**Alcohol**	
3.516	632	636	2-methyl-1-propanol	41/43/74
5.696	739	738	2-methyl-1-butanol	41/57/70
10.404	857	859	*Z*-3-hexen-1-ol	41/67/82
10.960	868	863	*Z*-2-hexen-1-ol	41/57/82
11.108	870	869	1-hexanol	43/56(69
16.924	967	968	*Z*-hept-4-enol	41/81/96
17.260	972	973	1-heptanol	43/56/70
21.275	1030	-	*Not identified*	43/57/98
21.487	1033	1033	Benzyl alcohol	77/79/108
24.009	1069	1055	*E*-2-octen-1-ol	41/57/68
24.226	1072	1068	1-octanol	41/56/70
27.038	1109	1114	Phenylethyl alcohol	65/91/122
30.837	1165	1165	[1S-(1α,2α.,5β)]-Cyclohexanol, 5-methyl-2-(1-methylethyl)	71/95/123
31.386	1165	1165	Levomenthol	71/81/95
			**Aldehyde**	
3.925	663	665	3-methyl-butanal	44/58/71
4.055	670	671	2-methyl-butanal	29/41/57
7.816	797	798	Hexanal	44/56/82
10.226	854	853	2-hexanal	41/69/83
22.159	1043	1043	Benzeneacetaldehyde	65/91/120
23.218	1058	1062	*E*-2-octenal	41/55/70
26.532	1101	1102	Nonanal	41/57/98
33.733	1203	1203	Decanal	43/70/112

			**Ester**	
19.762	1006	1009	*Z*-3-hexen-1-ol, acetate	43/67/82
20.221	1013	1012	Hexyl ester acetic acid	43/56/84
			**Ketone**	
7.459	789	787	2-hexanone	43/58/100
12.254	889	889	2-heptanone	43/58/71
18.338	986	986	6-methyl-5-hepten-2-one	43/69/108
18.603	990	991	2-octanone	43/58/71
25.678	1090	1091	2-nonanone	43/58/71
29.277	1143	1143	(1S)-1,7,7-trimethyl-bicyclo[2.2.1]heptan-2-one	41/95/152
39.817	1292	1291	2-undecanone	43/58/71
			**Terpene**	
21.202	1029	1031	Limonene	68/93/136
45.283	1375	1376	α-cubebene	105/119/161
45.363	1377	1377	Copaene	105/119/161
47.192	1403	1403	Longifolene	94/161/189
			**Other**	
13.122	903	901[c]	Methoxy-phenyl-oxime	77/133/151

[a]RIcalc: retention indices calculated from C8 to C20 n-linear alkanes with TRB-5MS capillary column.
[b]RIlit: retention indices reported in the literature for TRB-5MS capillary column or equivalent.
Identification method: Compounds were identified by comparing the MS fragmentation pattern with those of STD and mass spectra database performed using NIST Standard Reference Database 69, PubChem Compound Summary and ChemSpider database. Considering fit and retrofit values >80 %.
[c]Chung et al. (2021)

Table S2 Relative importance of the treatment (olives inoculated with *Colletotrichum acutatum* or *Aureobasidium pullulans*, or *A. pullulans* + *C. acutatum*) and time of microbial interaction (2, 8 and 16 days), on volatile composition as revealed by permutational multivariate analysis of variance (PERMANOVA).

Variable	DF	SS	F-statistics	R^2	P value
Treatments	3	1.2440	5.264	0.130	0.001
Sampling time	1	4.0262	51.116	0.420	0.001
Residuals	55	4.3321		0.451	
Total	59	9.6023		1.000	

Legend: DF - degrees of freedom; SS - sum of squares

References

Chung, M.; Cheng, S.; Lin, C.; Chang, S. Profiling of volatile compounds with characteristic odors in *Bambusa oldhamii* shoots from Taiwan. *BioResources* **2021**, 16(3), 5901-5914.
